# The Novel CarbaLux Test for Carbapenemases and Carbapenem Deactivating AmpC Beta-Lactamases

**DOI:** 10.3389/fmicb.2020.588887

**Published:** 2020-11-23

**Authors:** Hans Rudolf Pfaendler, Hans-Ulrich Schmidt, Heike Freidank

**Affiliations:** ^1^Department of Chemistry, Ludwig-Maximilians University, Munich, Germany; ^2^Department of Medical Microbiology, München Klinik gGmbH, Munich, Germany

**Keywords:** antibiotic resistance, rapid CarbaLux test, fluorescent carbapenem, carbapenemase, plasmidic resistance, AmpC beta-lactamase, antibiotic trapping, inactivation mechanism

## Abstract

**Objectives:**

To evaluate the rapid phenotypic CarbaLux test for routine diagnostics in the medical laboratory in a proof of concept study.

**Methods:**

isolates of Gram-negative bacteria suspicious for carbapenem resistance including *Enterobacterales* (67), *Pseudomonas* (10), *Acinetobacter* (5), and *Stenotrophomonas* (1) species, collected between 2016 and 2018 from in-patients, were tested for carbapenemase activity using a novel fluorescent carbapenem. When subjected to extracted bacterial carbapenemases its fluorescence disappears. All bacteria to be tested were cultured on Columbia blood agar and few on other commercial media. MALDI TOF MS, molecular assays, automated MIC testing, and in part, agar diffusion tests served to characterize the isolates. For comparison, few selected bacteria were also investigated by prior phenotypic tests for carbapenemase detection.

**Results:**

Under UV light, the CarbaLux test allowed a rapid detection of 39/39 carbapenemase-producing bacteria, including 15 isolates with OXA carbapenemases (e.g., OXA-23, OXA-24/40-like OXA-48-like or OXA-181). Several isolates had low MICs but still expressed carbapenemases. Among *Enterobacter* spp., it detected six strains with hyper-produced AmpC beta-lactamases, which deactivated carbapenems but were not detectable by prior rapid phenotypic assays. An unexpected high carbapenemase activity appeared with these enzymes. They were identified as AmpC variants by inhibition with cloxacillin.

**Conclusion:**

Other than prior rapid phenotypic assessments for carbapenemases, which use secondary effects such as a change of pH, the inactivation of the fluorescent carbapenem substrate can be visualized directly under UV light. The new test works at 100 to 200-fold lower, therapy-like substrate concentrations. It takes advantage of the high substrate affinity to carbapenemases allowing also the detection of less reactive resistance enzymes via a trapping mechanism, even from bacteria, which might appear unsuspicious from initial antibiograms. The novel fluorescence method allows simple and safe handling, reliable readings, and documentation and is suitable for primary testing in the clinical laboratory.

## Introduction

One of the most disastrous environmental changes of the early 21st century is the insidious alteration of human intestinal bacteria. Bacteria have learned to overcome the pressure exerted by over-use of antibiotics and within the last two or three decades resistance towards antibiotics, in particular beta-lactam antibiotics, emerged. Today, a considerable part of the human population and of domestic and wild animals harbors intestinal bacteria resistant to penicillins and cephalosporins (Economou and Gousia, 2015). Recently, also carbapenem resistance progressed. The responsible *Enterobacterales* are often associated with non-susceptibility to most other antibiotics including quinolones. They pose a threat to human life and, in case of infections, offer few options for successful therapy (Codjoe and Donkor, 2018).

Yet, this phenomenon remains mostly unrecognized within the healthy population, because colonized carriers are non-symptomatic and in fact, the resistant bacteria behave like their wild type relatives carrying out physiological functions; e.g. they participate in the digestion of food, adjust the equilibrium with other bacteria, and regulate the pH in the gastrointestinal tract etc.

However, in hospital settings these microorganisms can proceed from the gut of the host to the bloodstream and cause severe sepsis. They can colonize neonates, other immunocompromised patients at risk for invasive infection, or even members of the hospital staff. Therefore, the early detection of multi-resistant gram-negative bacteria is paramount (Hrabak et al., 2014).

There are several mechanisms contributing to acquired carbapenem resistance of gram-negative bacteria. Besides changes in the cell wall, determining the decrease of the influx or the increase of efflux of antibiotics, (Davin-Regli and Pagès, 2015) formation of hydrolyzing enzymes is the predominant contribution to resistance against carbapenems (Queenan and Bush, 2007).

Carbapenemases show a great structural variety. Most investigated are VIM, NDM, IMP, KPC, OXA-48-like beta-lactamases and a few others. They are either metallo or serine type beta-lactamases.

From all class D beta-lactamases, including OXA penicillinases, the OXA-48-like carbapenemases are distinct, due to their higher and broader catalytic efficiency. In Europe, they are the most observed among all classes of carbapenem hydrolyzing enzymes. They occur in manifold modifications and vary often only by the substitution of a single amino acid. Currently, more than 400 variants possessing carbapenemase activity have been described (Queenan and Bush, 2007; Antunes et al., 2014; Bush, 2018). Because of relatively low enzymatic reactivity and expression they may often remain undetected in microbiological laboratories (Poirel et al., 2012; Hrabak et al., 2014).

A second scarcely investigated but progressing (Jacoby, 2009; Thomson, 2010) type of carbapenem resistance arises from molecular Class C beta-lactamases (AmpCs), (Philippon et al., 2002) which also show a broad structural diversity. Around 800 variants have already been identified (Bush, 2018). Their genes are located in the chromosomes of several members in the family of Gram-negative bacteria but the production of the enzymes was largely depressed in the wild-type bacteria. With the exposition to beta-lactam antibiotics, mutants with stable hyper-production have emerged. The mode of expression varies from usually stable repression (e.g., in *Escherichia coli*), to inducible production (e.g., in *Enterobacter cloacae, Klebsiella aerogenes, Citrobacter freundii, Serratia marcenses*, and other species). Constitutive high-level production of chromosomally encoded AmpCs often results from mutations in regulatory genes.

Over the years, *bla*AmpC genes have been mobilized by transfer into plasmids (Jacoby, 2009). High level enzyme expression often arises due to a lack of genetic repression and by the presence of effective promoters on plasmids. The gene transfer occurred not only among the original ancestors, e.g., in *E. cloacae* (Peymani et al., 2016) and *K. aerogenes*, (Gazouli et al., 1996) but also into other genera including, *Klebsiella pneumoniae, Salmonella* spp., and *Proteus mirabilis* that were initially devoid of this particular genetic information, (Philippon et al., 2002) or into *Escherichia coli*, having a (normally) silent chromosomal *bla*AmpC gene. Most investigations were based on such isolates because their effective plasmidic gene location was unambiguous.

Earlier bacteria with chromosomal location and relatively low expression of *bla*AmpC genes were susceptible to carbapenems and low-level resistant to cefoxitin (Polsfuss et al., 2011). In contrast, the enzyme expression of particular plasmidic genes of *Enterobacter cloacae* can be increased up to 95-fold (Jacoby, 2009). Over-production of plasmid-mediated AmpC (pAmpC) beta-lactamases is associated with constitutive multi-resistance (Gazouli et al., 1996; Bush, 2018) and of concern because of the easy transfer of resistance genes between bacterial species and the association with hospital outbreaks (Philippon et al., 2002; Rensing et al., 2019). However, this might also hold for chromosome-mediated resistance: In a Taiwanese hospital, 41 cases of bloodstream infections caused by *E. cloacae* (Ku et al., 2019) were impeded by a high participation (74 %) of plasmidic *bla*AmpCs, most of a non-clonal origin. This suggests a frequent gene transfer from chromosome to plasmid within this genus.

In summary, essentially all Gram-negative pathogens can host chromosomally mediated (cAmpC) or acquired (pAmpC) beta-lactamases. When over-produced they contribute to or imply carbapenem resistance, (Noyal et al., 2009; Bush, 2018) particularly in connection with reduced cell wall permeability (Raimondi et al., 1991; Bradford et al., 1997) of the bacterial pathogens.

When focusing on clinical *E. coli* isolates, the prevalence of pAmpC resistance can differ worldwide. It was as low as 0.12 % in Denmark but up to 45.5 % in India. In 2019, in Egypt, 2.4 % of *E. coli* and 10.5 % of *K. pneumoniae* clinical isolates were plasmidic *bla*AmpC carriers (Rensing et al., 2019). In Germany, AmpC, combined with reduced cell wall permeability, is the predominant factor for carbapenem resistance of *E. cloacae* and *K. aerogenes*, (Lübbert, 2016) which have also been described as the third and fifth leading causes for recent infections caused by *Enterobacterales* in France (Davin-Regli and Pagès, 2015). *Enterobacter* spp. can be associated with the dissemination of actual epidemic plasmids bearing most prevalent resistance genes and expressing new beta-lactamases or carbapenemases. Today, multiple combinations of beta-lactamases within one organism are common and can make phenotypic identification difficult. Therefore, AmpC resistance goes undetected in most clinical laboratories (Perez-Perez and Hanson, 2002; EUCAST, 2017a). Outbreaks were frequently associated to AmpC beta-lactamase producing bacteria (Philippon et al., 2002; Jacoby, 2009; Davin-Regli and Pagès, 2015) and high mortalities were observed in Britain among infected persons who received therapy with cephalosporins (Livermore and Woodford, 2006) or worldwide among patients treated with meropenem (Tamma et al., 2013; Harris et al., 2016). In a three years cohort study, from all bacterial infections, bacteremia by AmpC producing *Enterobacterales* was the second-highest risk factor for inappropriate therapy and patient mortality, even higher than that for MRSA bacteremia (Picot-Guéraud et al., 2015). In summary, AmpC enzymes are largely underestimated (Philippon et al., 2002; Jacoby, 2009) and pose a vital danger because their activity contributes to the effect of other beta-lactamases or of the other resistance factors (Noyal et al., 2009).

### Methods for Carbapenemase Detection

#### Susceptibility Testing

For over fifty years, antibiotic therapy was to be oriented towards MIC testing and automated systems have become established in the clinical laboratory. However, susceptibility testing, while adequate for less complicated organisms, often fails with new pathogens that express multiple resistance mechanisms and produce multiple types of beta-lactamases (Perez-Perez and Hanson, 2002). In particular, *K. pneumoniae* and *E. coli* producing plasmid-mediated carbapenemase were associated with serious errors of false susceptibility in routine inhibition tests (Black et al., 2005; Queenan and Bush, 2007). But also other isolates, producing VIM-carbapenemases, provided carbapenem MICs around the clinical breakpoints (Asthana et al., 2014) or were as low as 0.125 μg/ml (Psichogiou et al., 2008) so that laboratory reports of false-positive susceptibility to carbapenems can lead to false therapy and be potentially fatal.

In susceptibility testing *low inocula* are used, which may not always correspond with the *in-vivo* situation. It was found that *K. pneumoniae* isolates with borderline MICs provided a marked inoculum effect, absent for their wild type relatives (Adler et al., 2015). A key point is that *clinical resistance* often arises only during antibiotic therapy by induction or the selection of few mutants, (Jacoby, 2009) and may go unrecognized through early MIC testing (Thomson, 2010; Hrabak et al., 2014). In fact, most strains with pAmpC enzymes have been isolated from patients after several days in hospital (Philippon et al., 2002). To improve reliability, lower epidemiological cut-off values for the detection of carbapenem resistance were recently proposed by the European Committee on Antimicrobial Susceptibility Testing (EUCAST) (EUCAST, 2017b).

In consequence, susceptibility testing to carbapenems should be complemented by a direct test for carbapenemase activity.

#### Molecular Tests or PCR Assays

When cultured on agar plates, methicillin resistant *Staphylococcus aureus* (MRSA) isolates can be reliably identified by amplification of mecA and mecC gene and its variants (Aydiner et al., 2012; Hirvonen et al., 2012). In contrast, resistant Gram-negative bacteria are far more difficult to assess. They can exhibit more than 2700 confirmed beta-lactamases (Bush, 2018) and molecular tests are limited to the detection of relatively few congeners, as only known and pre-selected carbapenemase genes can be addressed. Consequently, new variants might be missed (Kaase et al., 2012).

The value of such molecular methods is rather due to their high sensitivity and reliability to detect and identify *particular* resistance genes. Naturally, they neither provide an estimate of the level of gene expression nor of the reactivity of the investigated enzymes. As for the ubiquitous AmpC found in several classes of Gram-negative bacteria, a molecular test would always provide a positive result, independent of the phenotypic resistance. Consequently, in organisms that produce an inducible chromosomal *bla*AmpC such testing is unnecessary because the organism identification is indicative of AmpC production (Thomson, 2010). On the other hand, molecular identification of the most common plasmid-located *bla*AmpCs (*bla*CMY, *bla*DHA, *bla*FOX, *bla*MOX, etc.) is possible, due to lack of significant homology with intrinsic *bla*AmpC genes. The main constraint, however, is the multiplicity of targets. Unfortunately, a commercial test is not available.

#### Phenotypic Test Methods

Phenotypic test methods are broader in scope but also face a particular problem of the versatile character of resistant bacteria: The amount and reactivity of carbapenemases vary largely between the individual strains and enzymes (Queenan and Bush, 2007). In particular, enzymes of low reactivity or at low level are difficult to detect.

In an actual summary (Ludgring and Limbago, 2016) and in multiple original research articles the current test methods were described, e.g., in Sun et al. (2017) where six assays were evaluated, all based on hydrolysis of carbapenem antibiotics. With all these procedures bacteria from agar plates were tested. Three methods required additional overnight culturing of an *E. coli* indicator strain and three investigated variants (pH based methods) were rapid assays with test results available on the same day, usually within ca. 2 hr of investigation. A positive test arises from a slight reduction by ca. 0.2 pH of the medium, when the extracted bacterial enzyme have hydrolyzed a part of imipenem. It is accompanied by a continuous shift in color tone of an acid-base indicator, which is compared to the color shade of a reference sample with bacteria but without imipenem. These tests use high inocula and imipenem concentrations of up to 6000 μg per mL. The investigation (Sun et al., 2017) provided very high sensitivities, except for many OXA-type carbapenemases (17–80 %) and none of the three rapid pH methods evaluated could identify AmpC in a panel of 52 isolates with imipenem MICs of 1–128 μg per mL. In contrast, two inhibition zone-based methods MHT (modified Hodge test) and THT (triton Hodge test) provided 7 and 10 positive results from isolates, which all harbored the plasmid located *bla*AmpC genes.

The pH based tests were subject to a multiple change of protocols. Major issues are the setting of a reliable cut-off point, i.e., an unfailing tone of color, inaccurate detection by the human eye, and interpretation of borderline results. Deviating test results were attributed to extra challenging bacteria (Österblad et al., 2016) and/or to the presence or absence of buffer or to different culture media (Lifshitz et al., 2016). Apparently, for many of the important OXA-carbapenemase producing bacteria, the pH based tests may lack substantial sensitivity, (Huang et al., 2014; Pasteran et al., 2015; Lifshitz et al., 2016; Sun et al., 2017). [Tan JBX, Goh SS. 2017; 27th European Congress of Clinical Microbiology and Infectious Diseases, Abstract No. EV0030] or selectivity (Huang et al., 2014; Lifshitz et al., 2016; Österblad et al., 2016). Moreover, they cannot detect carbapenem deactivation by over-produced AmpC beta-lactamases (Lifshitz et al., 2016; Sun et al., 2017). In view of the worldwide abundance, ability to disseminate, and the high patient mortality associated with bacteria harboring or co-harboring such enzymes, improved methods of assessment are needed urgently.

A universal test for carbapenemase should be reliable, specific, inexpensive, simple, rapid, broad, easily read, and documented, in view of the diversity of enzymes a goal hard to achieve in a single test method. It must also be highly sensitive to detect borderline resistance, likely expanding during therapy.

A promising solution was an enzymatic test mimicking the inactivation of carbapenem antibiotics during therapy when exposed to carbapenemase-producing bacteria. We present here the novel phenotypic CarbaLux^®^ test.

### Principle of the CarbaLux Test

**FIGURE 1:**
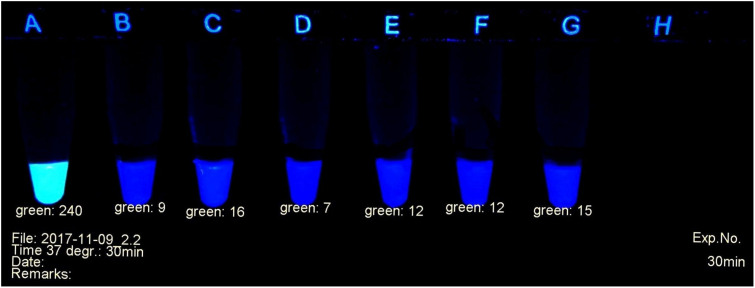
Example of carbapenemase assessment under UV light. **(A)** *E. coli* ATCC 25922; **(B)** and **(C)** *P. mirabilis* OXA-48 Nos. 29bl and 29ca; **(D)** *E. coli* NDM No. 21bl; **(E)** *K. pneumoniae* KPC; No. 11bl; **(F)** *A. baumanii* OXA-40 No. 25bl; **(G)** *E. ludwigii* hyper-producing AmpC No. 83bl.

A fluorescent carbapenem substrate (Pfaendler and Golz, 2016) served to detect the carbapenemases extracted from bacteria. Upon contact with active enzymes its fluorescence disappears, allowing to monitor the decay of the substrate under a laboratory UV lamp as shown in Figure 1. The nitrocefin test (O’Callaghan et al., 1972) used in many laboratories for the detection of beta-lactamases works with a similar principle. The difference is the decay of yellow-green fluorescence in the former versus the formation of red color in the latter during hydrolysis of the substrate. Additionally, the fluorescent probe is specific for carbapenemases.

## Materials and Methods

### The CarbaLux Test

#### Applied Culture Media and Substrates

The CarbaLux test was performed on strains grown on Oxoid Columbia Agar + Sheep blood (Product PB 5008A), Oxoid Columbia Agar + Sheep blood Plus (Product PB5039A), Oxoid Mueller-Hinton Agar (Product PO5007A), Mast CHROMagar ESBL (Product 201470), Mast CHROMagar mSuperCARBA (Product 201473), Oxoid Brilliance ESBL Agar (Product PO5202), and Oxoid Brilliance CRE Agar (Product PO1226). The blood agar medium (PB 5008A) was used in all reported tests. The other media were applied in fewer cases when available or intended for another testing in daily practice.

*Reference strains E. coli* CCUG 58543 (pAmpC CMY-2) and *K. pneumoniae* CCUG 58545 (pAmpC DHA) were obtained from Culture Collection University of Gothenburg, Sweden.

*Fluorescent carbapenem substrate*, aliquoted in polypropylene tubes (Eppendorf Safe-Lock Tubes, PCR clean, Product 0030 123.328), was provided by CarbaLux^®^ GmbH, 82544 Egling, Germany. Similarly, tubes with substrate and cloxacillin (CLX) were available. The extraction medium consisted of an aqueous buffer solution (pH 7.4) containing a non-ionic tenside and was obtained from the same supplier.

#### Set-up for Detection of Fluorescence Levels

The progress of the reaction was investigated in a UV cabinet with exclusion of daylight. UV light of 312 nm was produced by a modified laboratory lamp (Herolab UV-B 6W, Product 2950510) used as a transilluminator. After appropriate time intervals, the tubes were photographed (Figure 1) with a mounted Logitech C 920 webcam. The individual levels of the recorded fluorescence were determined using a red-green-blue photo program. Green values were suitable to estimate the amount of non-hydrolyzed substrate. They served as criteria to determine the presence or absence of a bacterial carbapenemase. Typically, during a positive reaction, the values decreased from ca. 240 (bright yellow-green fluorescent) to ca. 20 (non-fluorescent, dark gray tube). The camera settings were adjusted to these values and kept throughout all experiments. A test considered positive arose, when the yellow-green fluorescence had decreased below the value of 100 (see Figure 1). Alternatively, an ordinary UV lamp containing a 312 nm tube also can serve to visualize the decay of the fluorescence in the dark.

The following protocol for the CarbaLux test was applied:

#### Culturing of Bacteria and Enzyme Extraction

After fractional loop inoculation the agar plates were incubated for 15–18 h at 37°C. Bacteria from a sparse growth area or single colonies were collected to a full 1 μL loop (approximately 5 to 8 mg of wet bacteria) and suspended in CarbaLux^®^ extraction medium (100 μL) in 1.5 mL tubes, containing the lyophilized fluorescent substrate or its composition with cloxacillin. Up to eight samples were prepared in parallel. The pellets were completely dispersed manually, and the tubes were locked followed by short vortexing. They were left at room temperature allowing to the last prepared sample an additional extraction period of 15 min. Subsequently, the tubes were centrifuged for 3 min at 13’500 rpm and were ready for the first fluorescence measurement (time zero).

The centrifugation step is optional for cultures from blood agar or Mueller-Hinton agar but mandatory with dyed bacteria from chromogenic media (Mast CHROMagar or Brilliance agar media). To standardize the protocol, all samples were centrifuged prior to the first UV measurement.

#### Enzymatic Hydrolysis and Measurement of Fluorescence Levels

Simultaneously, up to eight tubes were investigated under UV light. A picture was taken at time zero, i.e., when the last preparation had been allowed an extraction period of 15 min. In order to achieve the highest possible reactivity, the tubes were kept in a heating block at 37°C for 120 minutes in total. After 30, 60, and 120 min snapshots were taken, after allowing the tubes (for 1 to 2 minutes only) to get room temperature. They remained only shortly exposed to UV light. A sample with carbapenemase-negative *E. coli* ATCC 25922 or *P. aeruginosa*. ATCC 27853 was co-investigated in each run.

### Susceptibility Testing and Molecular Assays

Bacterial strains were identified by MALDI-TOF mass spectrometry (Microflex^®^) Bruker Daltonics GmbH, Bremen, Germany. Routine susceptibility testing was performed by Vitek^®^ 2 (bioMérieux GmbH, Nürtingen, Germany). To confirm results found in cases of low MICs around the clinical cutoff, gradient diffusion tests were done by MIC Test Strips (Liofilchem/bestbion dx GmbH, Köln, Germany). Antibiograms of isolates were interpreted according to CLSI breakpoints. All carbapenem non-susceptible strains, selected for the CarbaLux test, were investigated for carbapenemase genes at least by one of the following molecular assays: eazyplex^®^ SuperBug CRE (Amplex Biosystems GmbH, Giessen, Germany) or Xpert^®^-Carba-R (Cepheid GmbH, Frankfurt, Germany). Addressed carbapenemase genes were *bla*KPC, *bla*NDM, *bla*VIM, *bla*IMP-1, *bla*OXA-48, *bla*OXA-23, *bla*OXA-24/40, *bla*OXA-58, and in one instance *bla*OXA-181. A 0.5 mM solution of ferrous sulfate (for removal of fluorescence, frequently associated with *P. aeruginosa*) was freshly prepared by dissolving FeSO_4_ heptahydrate in 2.5 mM H_2_SO_4_.

### Spectroscopic Carbapenem Hydrolysis Assay

#### Beta-Lactamase Reactivity of Selected Bacteria

The aim of these investigations was to compare and correlate positive CarbaLux test results with those of a proven assay (Nagano et al., 1999) From a selection of four isolates Nos. 2, 16, 26, and 79 the activities of crude protein extracts towards the fluorescent substrate, imipenem (IPM), meropenem (MEM), and nitrocefin were measured in a Varian Cary 50 Scan spectrophotometer. For protein extraction, 15.0 mg of wet bacteria were harvested from an overnight culture on Columbia agar with 5 % sheep blood (Oxoid), suspended in phosphate buffer pH 8 (750 μL) in a 1.5 mL plastic tube, and sonicated at 0°C for 10 min. A Bandelin SONOPULS GM 2070 and a 2 mm tip (supplier Bandelin Electronics, Heinrichstrasse 3–4, D-12207 Berlin, Germany) were used at 30 % cycle and 30 % power settings. The initial turbid solution became transparent after ca. 7 min. The tube was centrifuged and the supernatant stock solution kept at 3°C. Variable volumes of fresh stock solution (e.g., 50 μL, corresponding to 1.0 mg of wet bacteria) were diluted in phosphate buffer pH 8 to a final volume of 600 μL in an appropriate UV cell, allowing a complete reaction of 20 μg substrate within an investigation period of ca. 1 to 6 h at 30°C. The decay at UV maximum absorption of the carbapenems IPM, MEM, or fluorescent carbapenem and the increase at 490 nm with the cephalosporin nitrocefin were recorded at different time points. The initial reactivity was obtained by plotting extinctions vs. time, considering the early phase of the decay. Relative reactivities refer to μg of hydrolyzed substrate per hr and per mg of (wet) bacterial cells. Nitrocefin served for setting up this method and as a standard due to the visible color change.

### Inhibition of Nitrocefin Hydrolysis by Carbapenems

Bacterial extracts from isolate No. 79 were obtained by sonication as above-mentioned. The rates of the nitrocefin hydrolysis were determined by spectroscopic assay at 1: 6000 dilution of the extract solution (corresponding to 3.3 μg of wet bacteria investigated per mL) in phosphate buffer pH 8 at 30°C. IC_50_ were determined from investigations at five different inhibitor concentrations as described (Goessens et al., 2013).

### Additional Investigations by Carbapenem Inactivation Method (CIM) and by the Carba NP-Direct Test (CNPt-direct)

Selected isolates were investigated by CIM as reported (Gutiérrez et al., 2019). A 10 μg imipenem (IPM) disk was added to a bacterial suspension in 400 μL water and incubated for 2 hr at 35°C prior to the investigation by agar diffusion testing at 35°C. The indicator strain was *E. coli* ATCC 25922. Accordingly, the CIM was repeated with meropenem (MEM) as reported (Sun et al., 2017). The inactivation period was extended to 6 h at 35°C.

The Carba CNPt-direct test was performed as reported (Sun et al., 2017).

### Assessment by Double Disk Method for AmpC Inducibility

Selected isolates of were investigated by the Double Disk Method (Gupta et al., 2014). Mueller-Hinton plates, inoculated with the investigative isolates and disks with IPM (10 μg) as an inducer and supplemented disks (by adding appropriate amounts of 2 % aqueous solutions of the antibiotic to the commercial disk, in case of insoluble ceftazidime, of a 2 % solution in phosphate buffer pH 8) containing cefoxitin (250 μg), ceftriaxone (250 μg), ceftazidime (250 μg), and aztreonam (100 μg) or original cefotaxime disks (30 μg) were placed 15 mm or 20 mm apart and incubated at 36°C overnight. Distorted inhibition zones were interpreted as inducibility.

## Results

### CarbaLux Test Results

Molecular (PCR) identification was used as a gold standard. The investigated bacteria, many with reduced susceptibility to carbapenems, were classified into two categories depending on positive or negative results of the CarbaLux test and the corresponding results are presented, respectively, in Tables 1, 2. The data on *Enterobacter* spp. and other AmpC producing strains are presented separately in Table 3 because the expression of AmpC in those strains was found to inactivate the fluorescent carbapenem in the CarbaLux test. Their molecular investigations revealed the absence of eight carbapenemase genes. For comparison, the results of two control strains harboring acquired p-AmpC beta-lactamase were also included in Table 3. Their use as reference strains was recommended by EUCAST (EUCAST, 2017a).

**TABLE 1:**
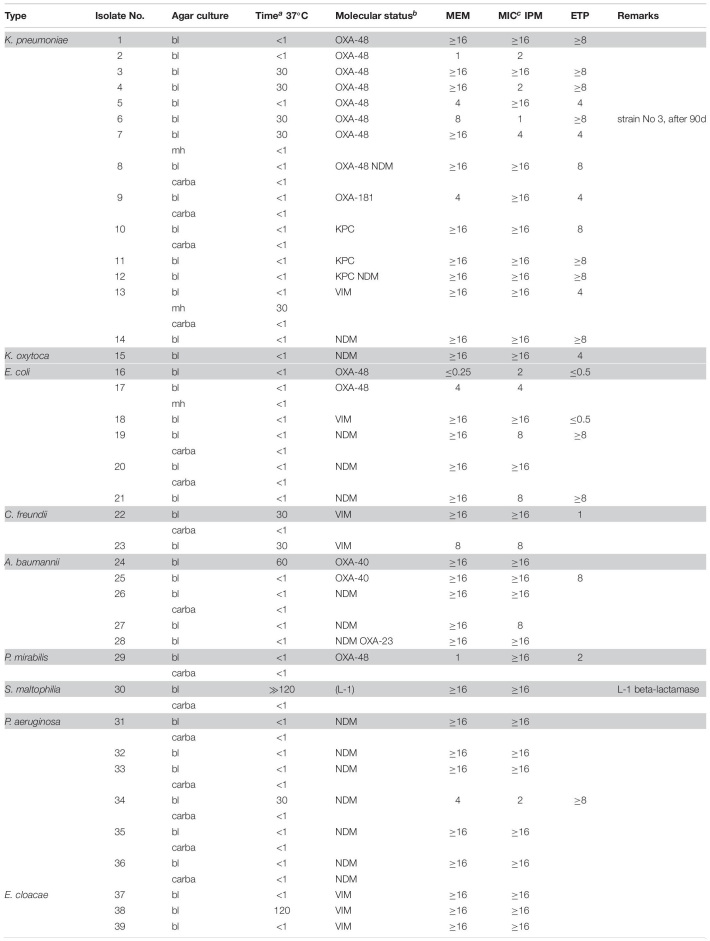
CarbaLux positive bacterial isolates.

*bl: Bacteria from Oxoid Columbia agar SB; mh: Oxoid Mueller-Hinton agar; carba: Mast CHROMagar msuperCARBA a) Time to positive CarbaLux results: ≫120 fluorescence retained after 120 min, negative test; >120 fluorescence reduced after 120 min, negative test; 120 no fluorescence after 120 min (dark tube), positive test; <1 tube is dark after extraction period at RT (15–45 min), positive test. b) Molecular status according to eight addressed carbapenemase genes. c) MIC data from automated testing. MEM meropenem, IPM imipenem, ETP ertapenem.*

**TABLE 2:**
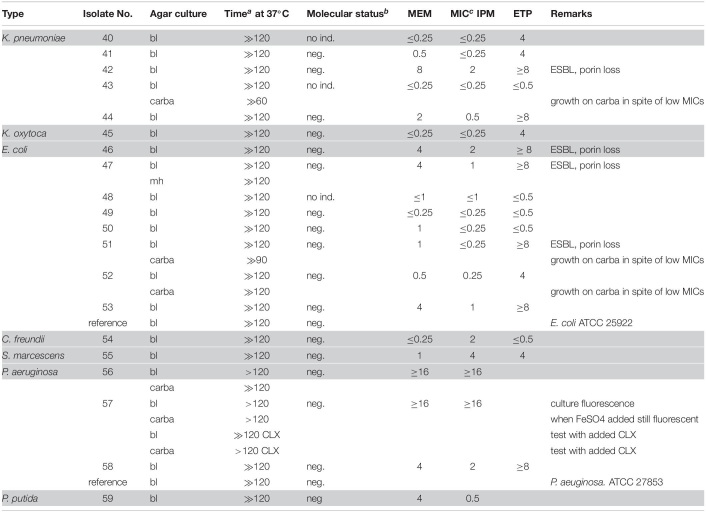
CarbaLux negative bacterial isolates.

*bl: Bacteria from Oxoid Columbia agar SB; mh: Oxoid Mueller-Hinton agar; carba: Mast CHROMagar msuperCARBA a) Time to positive CarbaLux results: ≫120 fluorescence retained after 120 min, negative test; >120 fluorescence reduced after 120 min, negative test; 120 no fluorescence after 120 min (dark tube), positive test; <1 tube is dark after extraction period at RT (15–45 min), positive test. b) Molecular status according to eight addressed carbapenemase genes. c) MIC data from automated testing. MEM meropenem, IPM imipenem, ETP ertapenem.*

**TABLE 3:**
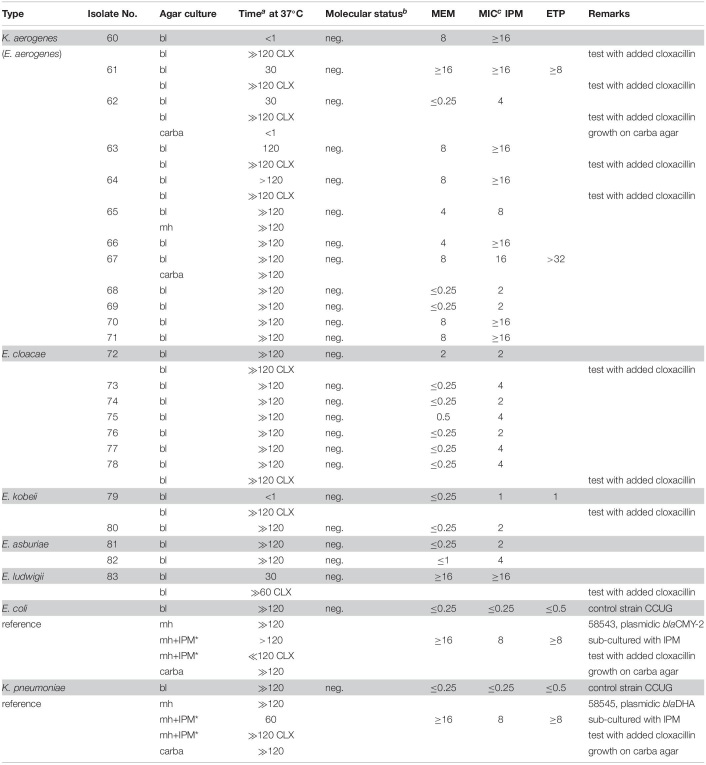
Isolates with potential AmpC beta-lactamase activity and negative molecular test for carbapenemase.

bl: Bacteria from Oxoid Columbia agar SB; mh: Oxoid Mueller-Hinton agar; carba: Mast CHROMagar msuperCARBA. a) Time to positive CarbaLux results: ≫120 fluorescence retained after 120 min, negative test; >120 fluorescence reduced after 120 min, negative test; 120 no fluorescence after 120 min (dark tube), positive test; <1 tube is dark after extraction period at RT (15–45 min), positive test. b) Molecular status according to eight addressed carbapenemase genes. c) MIC data from automated testing. MEM meropenem, IPM imipenem, ETP ertapenem. *) Bacteria were repeatedly sub-cultured in the presence of imipenem.

The relative carbapenemase reactivity was expressed (Table 1) by the time required, to provide a dark tube at elevated temperature (37°C). 30 out of 39 isolates of Group 1 including an *S. maltophilia* isolate with inducible carbapenemase (No. 30 carba) gave a rapid positive result, i.e., a dark tube, already at room temperature within the prior enzyme extraction period of 15 to 45 min. 38 out of 39 isolates were positive after 60 min at elevated temperature (37°C). Only one VIM positive isolate (No. 38 bl) took 120 min for complete reaction.

The unexpected low MIC values of bacteria, found in Table 1 (e.g., Nos. 2 and 16) could arise from low expression of carbapenemase and/or the presence of hetero-resistant sub-populations (Pournaras et al., 2010) which were accounted by PCR, but not by MIC testing. Low MICs are typical for particular *bla*KPC and *bla*OXA-48 carriers.

Several isolates of Table 2 provided MICs higher than CLSI breakpoints, suggesting the presence of beta-lactamases and/or other mechanisms. All strains tested negative for carbapenemase, with preservation of yellow-green fluorescence. With non-resistant *E. coli* ATCC 25922 or *P. aeruginosa* ATTC 27853 the fluorescence of the carbapenem substrate remained stable at least for 15 additional hours at room temperature.

Most isolates of Table 3 had MICs higher than the CLSI breakpoints, suggesting the presence of beta-lactamase and/or other mechanisms of resistance. Six out of 24 tested *Enterobacter* spp. (Nos. 60 bl, 61 bl, 62 bl, 63 bl, 79 bl, and 83 bl) gave a positive CarbaLux test. Cloxacillin (CLX) effectively inhibited their enzymatic reaction. When repeatedly sub-cultured at high inoculate in IPM-supplemented MH broth or MH agar, the MICs of both reference strains CCUG 58543 and CCUG 58545 with acquired AmpC enzymes had increased from ≤ 0.5 over the resistance breakpoint, to ≥ 8, whereas in the CarbaLux test only the inducible DHA strain CCUG 58545 became positive.

The unexpected high MICs of several isolates (e.g., Nos. 42 and 56) in Table 2 or (e.g., No. 71)in Table 3 could arise from an increased *bla*AmpC and/or *bla*ESBL expression, the presence of more than one beta-lactamase, (Perez-Perez and Hanson, 2002) from low permeability of the cell walls for antibiotics or an active efflux mechanism (Davin-Regli and Pagès, 2015).

In summary, the large and random span of MICs in Tables 1–3 supports the view that routine susceptibility testing alone cannot reliably detect all carbapenem inactivation mechanisms. This agrees with similar observations (Perez-Perez and Hanson, 2002; Tenover et al., 2006; Poirel et al., 2012; Harino et al., 2013) and demonstrates that special tests are required (Asthana et al., 2014).

#### Sensitivity and Specificity of the CarbaLux Test

The CarbaLux test detected all acquired carbapenemases in the tested isolates and of intrinsic L-1 beta-lactamase (Okazaki and Avison, 2008) of *S. maltophilia* No. 30 carba (Table 1) and when the hyper-producing AmpC isolates were excluded, the assay yielded no false-positive results with carbapenemase non-producing isolates (Tables 2, 3), indicating 100 % sensitivity and specificity.

#### AmpC and Inhibition by Cloxacillin

Six out of 24 tested *Enterobacter* or *K. aerogenes* isolates without molecular evidence for a carbapenemase gene (Table 3), gave a positive CarbaLux test. When repeated with added cloxacillin, a potent and specific inhibitor of AmpC, (Ingram et al., 2011) the reaction of the fluorescent carbapenem was inhibited and the six tests became negative. The span of the reactivity and the variable MICs of the said six isolates of Table 3 largely conform to those of Table 1 bacteria with classical carbapenemases.

#### Correlation of Reactivity by Spectroscopic Investigation

After finalizing the CarbaLux tests, it was still questionable to what extent the results would agree with the behavior of clinically used carbapenems. Because the fluorescence method cannot assess non-fluorescent substrates, hydrolyses of imipenem (IPM), meropenem (MEM), the fluorescent carbapenem, and nitrocefin were investigated in parallel in a UV spectrometer as previously reported (Nagano et al., 1999; Poirel et al., 2012). Crude protein extracts from selected isolates were used. The relative reaction rates (Table 4) allowed a conclusive comparison. Extracted NDM isolate No. 26 bl revealed a broad profile of specificity. With the serine-type beta-lactamases extracted from isolates 2 bl, 16 bl, and 79 bl, imipenem was the best (carbapenem) substrate, whereas with meropenem very low reaction rates at UV concentrations became apparent. Again, in this investigation the carbapenemase activities of the OXA-48 (No. 16) and AmpC (No. 79) extracts towards MEM appeared similar. Both isolates were susceptible (MIC ≤ 1) to MEM (Tables 1, 3). Understandably, the low enzyme concentrations investigated in 1 cm UV cells provide low reactivity. As the concentrations of the extracted enzymes could not be determined, exact kinetic parameters are not reported.

**TABLE 4:** Relative beta-lactamase activity of selected bacterial extracts^*a*^.

Type	Isolate number	Agar^*b*^	Molecular status	IPM	MEM	Fluorescent carbapenem	Nitrocefin
*K. pneumoniae*	2	bl	OXA-48	32	4	5.2	4800
*E. coli*	16	bl	OXA-48	14	1	1.1	1600
*A. baumannii*	26	bl	NDM	28	34	18	1100
*E. kobeii*	79	bl	negative	2.7	1.3	1.3	25000

*^a^Beta-lactamase activity was measured by UV spectrometer and refers to μg of substrate hydrolyzed per hr at pH 8.0 and 30°C by a crude extract of 1.0 mg of wet cells. Extraction was performed by sonication. ^b^Bacteria obtained from Oxoid Columbia agar SB (bl) culture.*

The low hydrolytic activity of OXA-48 and AmpC towards meropenem was reported earlier (Queenan et al., 2010; Antunes et al., 2014). However, even with the observed moderate *in-vitro* hydrolysis rate, smaller amounts of carbapenem agent still may be inactivated efficiently *in-vivo*, because the organism may produce exceptionally large periplasmic concentrations (up to 1 mM) of the beta-lactamase, (Walther-Rasmussen and Høiby, 2006) more than one hundred times the concentration of antibiotic in the cells. In conclusion, with regard to carbapenemase activity, results in Table 4 show that the fluorescent carbapenem closely resembled meropenem, qualifying the fluorescence test as a suitable tool to assess resistance patterns towards commercial carbapenem antibiotics, particularly meropenem.

#### Preferred Culture Media

The preferred culture medium was Oxoid Columbia Agar SB, which was successful with all investigated bacterial strains. Oxoid Mueller-Hinton agar, and Mast msuperCARBA were used only with a limited number of isolates and also appeared as suitable. Carbapenem susceptible *Pseudomonas* spp. and *Enterobacter* spp. frequently also grew on Mast mSuperCARBA, thus reducing the specificity of this plate for the detection of carbapenem resistance based on bacterial growth. Bacteria from this plate are stained and require a centrifugation step before the first measurement by the CarbaLux test.

*Inappropriate culture media* were Oxoid Brilliance CRE agar, which often did not provide enough bacteria within 15 to 18 hours of incubation. Two media developed for the isolation of ESBL producing bacteria (esbl and bresbl) provided a lower sensitivity, due to their content of inhibitors such as cloxacillin. MacConkey agar contains the fluorescent indicator Methyl Red, strongly interfering with the reading (results not shown).

*Stenotrophomonas maltophilia* carbapenemase is not addressable by commercial PCR gene tests. Isolate No. 30 (Table 1) gave a positive result only when grown on Carba agar media (containing active antibiotics).

*Fluorescence of Pseudomonas aeruginosa:* when present, initially interfered with the CarbaLux test. The natural phenomenon arises from blue fluorescent siderophors produced when the culture enters the stationary phase, (Sharma and Johri, 2003) if the medium is depleted of iron. The length of culturing time but also the type of medium was determinant. In particular, cultures from Mast ESBL agar and Oxoid Brilliance ESBL agar exhibited bacterial fluorescence frequently. Bacterial fluorescence was reduced and overcome by a short culturing period of 15 h at 37°C and by collecting single colonies or cells of an area of sparse growth from fresh cultures. Such harvested bacteria were not or only lightly stained when investigated in daylight and had no interfering fluorescence in UV light. The protocol was also applicable to all other investigated isolates in this study. Eventually and prior to investigations of *Pseudomonas aeruginosa* spp., the presence of culture fluorescence may be checked on the agar plate using a UV lamp. Non-fluorescent bacterial samples can then be located and collected. Culture fluorescence may also be determined from a bacterial suspension in 100 μL of extraction medium in a tube devoid of the substrate.

#### Additional Confirmation of Negative Test Results

Additional confirmation of negative test results was provided by the addition of 5 μL of carbapenemase LacBuster^TM^ -L (Eucodis Bioscience) into the tube after the last 120 min reading. Then, a rapid decay of the fluorescence within a few minutes at 37°C confirms that a still active substrate was investigated and the prior test was true negative. When fluorescence with *P. aeruginosa* spp. is observed the final (120min) test result can be checked by addition of low amounts of 0.5 mM ferrous sulfate solution (5 or 10 μL) into the vial, which suppresses the natural blue fluorescence instantaneously without affecting the yellow-green fluorescence of the carbapenem substrate notably. Thus, a misinterpretation of eventual culture fluorescence can be ruled out.

#### Safety

After the 120 min test phase, 99.99% of the bacteria (isolate No. 79) were killed, as determined by plate count methods. When stored at room temperature for 24 hr, no viable bacteria were found (data not shown). Save-Lock tubes secured further safety.

### Results from Other Phenotypic Carbapenemase Assays

#### Confirmation of Carbapenemase Activity by Carbapenem Inactivation Method (CIM)

**FIGURE 2:**
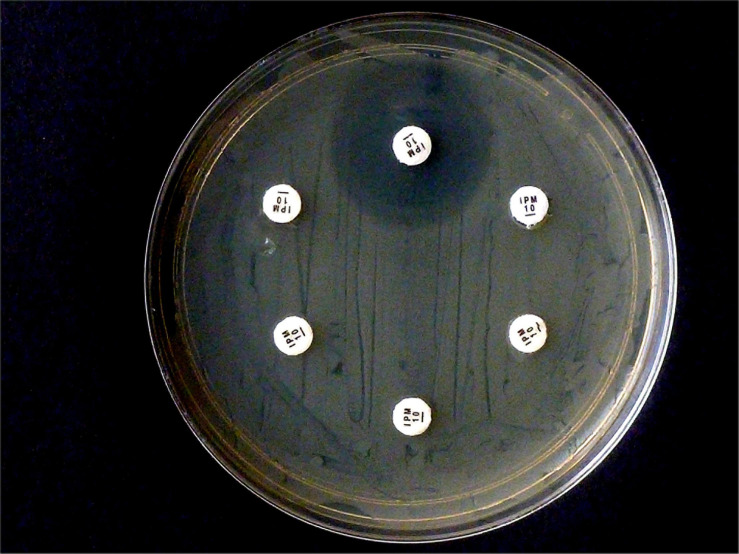
Results from carbapenem inactivation method CIM with imipenem. Tested isolates were (in clockwise sequence): *E. coli* ATCC 25922 (top), *E. coli* OXA-48 (No. 16), *E. kobeii* AmpC (No. 79), *K. aerogenes* AmpC (Nos. 60, 61, 62). A positive test arises when the antibiotic is hydrolyzed (inhibition diameter 6 mm) after exposure for 2 h at 35°C. Indicator strain was *E. coli* ATCC 25922.

Selected test results of the CarbaLux test were experimentally confirmed (Figure 2). The test was performed according to (Gutiérrez et al., 2019) with intact cells, suspended in water. Five isolates (one OXA-48 and four AmpCs) hydrolyzed 10 μg IPM rapidly and completely.

**FIGURE 3:**
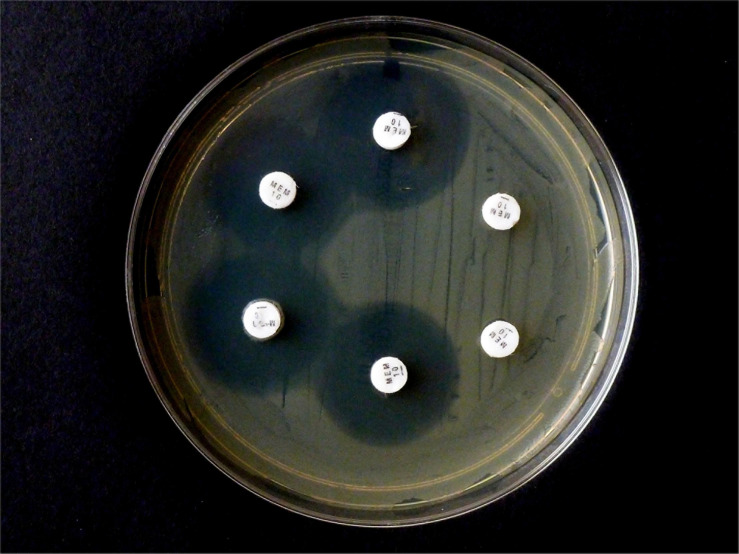
Results from carbapenem inactivation method CIM with meropenem. Tested isolates were (in clockwise sequence): *E. coli* ATCC 25922 (top), *E. coli* OXA-48 (No. 16), *E. kobeii* AmpC (No. 79), and *K. aerogenes* AmpC (Nos. 60, 61, 62). The MEM disks were kept in an aqueous bacterial suspension for 6 h at 35°C. Indicator strain was *E. coli* ATCC 25922.

In contrast, when repeating the CIM experiment with the same isolates and 10 μg MEM disks, *E. coli* OXA-48 No. 16 and *E. kobeii* No. 79 also gave a positive result after 6 hr at 35°C but the three *Klebsiella aerogenes* isolates Nos. 60, 61, and 62 tested negatives (Figure 3). The original CIM recommends a minimum reaction time of 2 hr at 35 °C.

#### Performance of the Carba NP-Direct Test (CNPt-direct)

**FIGURE 4:**
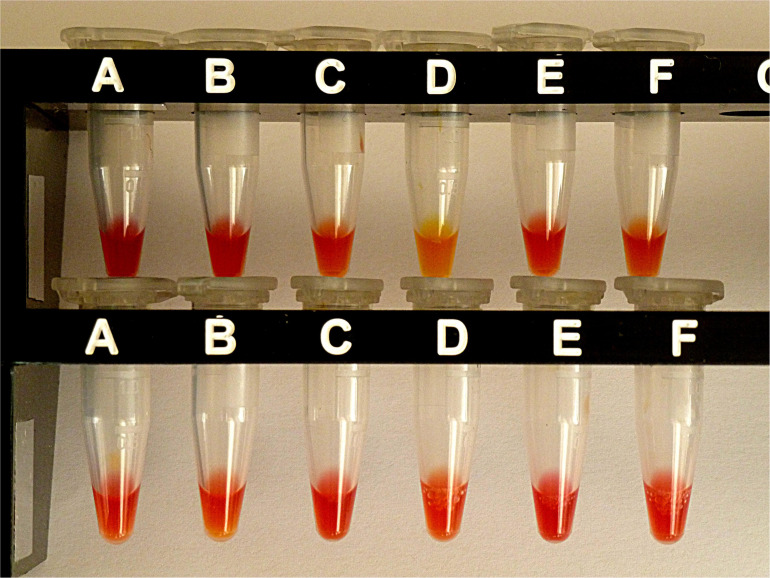
Results from CNPt-direct after exposure for 2 hr at 36°C. Matched pairs of isolate without and with IPM were in upper row **(A,B)** *E. coli* ATCC 25922, **(C,D)** *E. coli* OXA-48 (No. 16), **(E,F)** *E. kobeii* AmpC (No. 79); in lower row **(A,B)** *K. aerogenes* AmpC (No. 60), **(C,D)** *K. aerogenes* AmpC (No. 61), **(E,F)** *K. aerogenes* AmpC (No. 62). With a positive test the matched pair appears red/light-orange, red/dark-yellow, or red/light-yellow.

For comparison, the same selection of isolates was also investigated by CNPt-direct. With this method, enzymes are extracted by a non-ionic tenside. *E. coli* OXA-48 provided a weak positive reaction, whereas the four *bla*AmpC carriers and *E. coli* ATCC gave a negative test. Results are shown in Figure 4.

#### Performance of the CarbaLux Test in Water

Six CarbaLux positive isolates from Table 3 were investigated. When performing the test in water at 37°C accordingly, *E. coli* ATCC 25922 gave a negative result, retaining the level of fluorescence for more than 15 hr. Four *K. aerogenes isolates* Nos. 60–63 took 5 to 6 h to complete the decay. The reactivities of OXA-48 isolate No.16 and of AmpC isolates Nos. 79 and 83 were the same with full extinction of the fluorescence already after 30 min. The low substrate concentration and the density of the bacterial suspension corresponded to those of the CIM tests.

#### Inducibility of AmpC Beta-lactamase

CarbaLux positive isolates of Table 3, Nos. 60, 61, 62, 63, 79, and 83 were investigated by the Double Disk Method (Gupta et al., 2014). *K. pneumoniae* CCUG 58545 served as a reference. All six isolates provided a negative test result without any distorted inhibition zone, whereas the reference strain tested clearly positive.

#### Inhibition of Nitrocefin Hydrolysis by Carbapenems

Investigations of sonicated *E. kobeii* isolate No. 79 were performed according to (Goessens et al., 2013). Inhibition concentrations (IC_50_) for IPM, MEM and the fluorescent carbapenem were 0.1, 0.02, and 0.05 μM, respectively.

## Discussion

Given the horizontal transfer of plasmid bound carbapenemase genes to other bacteria, the detection of OXA carbapenemases is crucial. A highlight of the rapid CarbaLux test was that all 15 strains harboring *bla*OXA-48, *bla*OXA-40, *bla*OXA-23, or *bla*OXA-181 genes showed positive results with complete decay of fluorescence in ≤ 60 min at 37 °C (Table 1).

A second feature in this study was the rapid detection of six *Enterobacter* or *K. aerogenes* isolates Nos. 60–63, 79, and 83 (Table 3), due to AmpC beta-lactamase. In the past, this type of abundant (Rensing et al., 2019) and harmful (Picot-Guéraud et al., 2015) resistance to antibiotics including carbapenems, was laborious and time-consuming to assess by phenotypic tests (Ingram et al., 2011; Van der Zwaluw et al., 2015) and could not be detected by the rapid pH based methods (Sun et al., 2017). The reactions were inhibited by cloxacillin, characterizing the isolates as *bla*AmpC carriers, (Polsfuss et al., 2011).

The CIM tests investigate intact bacteria in water, without the extraction of the enzymes. The CIM variant using IPM was initially developed for the investigation of *P. aeruginosa* spp. (Gutiérrez et al., 2019). In agreement with the CarbaLux test results, this variant also provided positive test results for an *E. coli* OXA-48 and the four *bla*AmpC carrying *E. kobeii* or *K. aerogenes* isolates (Figure 2), irrespective of their MICs. Two further isolates Nos. 63 and 83 were also positive with no inhibition zone (plate not shown). On the other hand, after six hours of incubation, the original test with MEM (Van der Zwaluw et al., 2015; Sun et al., 2017) could detect, in addition to the OXA-48 strain No 16, only one AmpC isolate No. 79, whereof the MICs ≤ 1 were consistent with a permeable outer membrane (Figure 3). The strikingly different performance of the two established CIM variants to detect hyper-produced AmpC beta-lactamase may be due to the higher *in-vitro* reactivity of IPM. However, more likely, MEM did not fully pass the cell walls during the period of incubation and missed the periplasmic enzymes of particular bacteria. Investigations of IPM and MEM provided very different permeability coefficients for *E. cloacae* spp. (Cornaglia et al., 1995) and IPM exceptionally enters *Enterobacter* spp. via a porin channel different to that used by cephalosporins (Jones et al., 1997). Therefore, in this genus, the architecture of the cell wall might be of secondary importance for causing resistance to imipenem. With MEM, negative CIM test results have also been observed (Van der Zwaluw et al., 2015) of 14 *Enterobacterales* isolates with MICs > 8 and without evidence for a carbapenemase encoding gene.

Unlike the said six CarbaLux positive isolates from Table 3, the reference strains *E. coli* CCUG 58543 and *K. pneumoniae* CCUG 58545 with acquired *bla*AmpC genes were fully susceptible to three carbapenems with MICs ≤ 0.5. Both provided a negative CarbaLux test when cultured on regular media (Table 3), suggesting that the strains did not hyper-produce AmpCs, although CCUG 58545 was resistant to quinolone and aminoglycoside antibiotics. When these reference bacteria were sub-cultured several times at high inocula in MH broth and on agar plates supplemented with IPM, induction (Philippon et al., 2002; Jacoby, 2009) and/or selection (Black et al., 2005; Ingram et al., 2011) of resistant mutants occurred with an increase of carbapenem MICs. The mutated *K. pneumonia* DHA strain gave also a positive CarbaLux test. Subsequently, when re-cultured on IMP-free MH agar, the mutants lost the acquired carbapenemase activity, as observed by a negative CarbaLux test (data not shown). Likewise, resistance was induced in mutant strains of *E. cloacae* by IPM, leading to unstable cultures (Raimondi et al., 1991).

In the past, infections caused by chromosomal *bla*AmpC pathogens were curable by carbapenem therapy (Pai et al., 2004) but with the wide use of carbapenems, these bacteria were likely to become resistant (Ingram et al., 2011; Bush, 2018). In particular, *E. cloacae* was notorious for rapidly increasing MICs when sub-cultured in the presence of IPM and MEM (Neu et al., 1989; Jacoby, 2009). In a recent collection of the same species, (Peymani et al., 2016) a considerable part (20%) of pAmpC isolates was resistant against carbapenems and harbored the inducible (Jacoby, 2009) DHA-1-beta-lactamase. Our discovery of six isolates with effective AmpC (Table 3) seems just to confirm the earlier predictions. It might reflect a general advance of *Enterobacter* spp. towards multi-resistance. On the other hand, considering the lack of simple diagnostic methods, (Philippon et al., 2002; Ingram et al., 2011) such pathogens may have been more abundant than assumed, (de Gheldre et al., 1997). Particularly, isolates seemingly susceptible to MEM such as Nos. 62 and 79 may be malicious. Unstable cultures, e.g., from mutated *K. pneumoniae* GGUG 58545, may impede diagnosis and induction of DHA can affect the clinical outcome of therapy, when hetero-resistant pathogens are exposed to carbapenem antibiotics for days or weeks. Likely, the hidden resistance of such “wolves in sheep’s clothing” has largely contributed to their spread around the world.

When compared to other beta-lactam antibiotics, carbapenems are slow substrates for most carbapenemases, (Queenan et al., 2010; Poirel et al., 2012) consistent with high affinity but low turnover numbers (Walther-Rasmussen and Høiby, 2006; Bauvois et al., 2005). This makes detection by carbapenem hydrolysis difficult. Low enzyme levels – with particular *K. pneumoniae* isolates – they can be more than one hundred times lower for *bla*OXA-48 than for *bla*KPC carriers (Adler et al., 2015) – can create an additional obstacle for detection.

At low enzyme concentration, MEM is a potent inhibitor of carbapenemases and particularly of AmpC beta-lactamases (Goessens et al., 2013). This is also true for IPM and the fluorescent carbapenem, as found from our inhibition assay based on nitrocefin hydrolysis. The high inhibition activity can also explain the enigma of “false susceptibility”: When the resistance enzyme becomes inhibited by a minor part of the antibiotic, bacterial growth is prevented and a low MIC is observed, which may be falsely interpreted in that the investigated strain is devoid of carbapenemase. AmpC isolate No. 79, but also Nos. 2 and 16 may serve as examples. However, the delayed enzymes remain active. Surviving in aqueous solution for years at 5°C, as documented for the LacBuster^TM^ -L, particular carbapenemases are far more stable than the bacterial cells and far more stable than the antibiotics. Likely, the restored and persistent resistance enzymes also play an important role over the course of antibiotic therapy.

**FIGURE 5:**
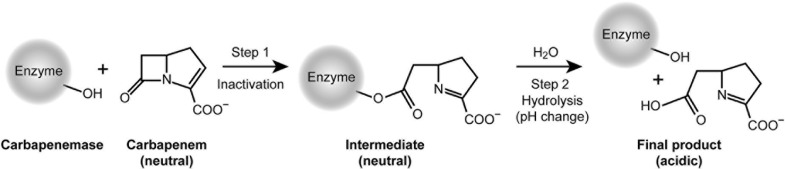
Mechanism relevant for the inactivation of carbapenem antibiotics and detection of carbapenemases.

The mechanism of carbapenem detection by phenotypic methods (Figure 5) occurs via two essential reaction steps: a rapid acylation of the enzyme (step 1), followed by a slow hydrolysis of the acyl-intermediate with concomitant restoration of the enzyme (step 2). According to Michaelis-Menten, the relevant kinetic parameter 1/*K*_*m*_ is a rough indicator for the substrate affinity to the enzyme and for the reactivity of step 1. In contrast, the catalytic efficiency *k*_*cat*_/*K*_*m*_ describes the reaction rate of step 2 and the overall conversion of the substrate into final product.

The CarbaLux test is unique, because it measures the first and always faster step, while opening the beta-lactam ring and reducing the fluorescence at the same time. This is not possible with the pH-based methods. Although their reaction also proceeds through step 1 with a rapid acylation and deactivation of IPM, they cannot provide a rapid visible effect because *step 1 occurs at constant pH*. These methods rely exclusively on a substantial turnover via steps 1 and 2, and respond only when the acidic final product is accumulated, providing a change of indicator color.

The results of kinetic measurements (Queenan et al., 2010) revealed that the rates of the two steps varied significantly among individual carbapenem antibiotics and enzymes. MEM reacted rapidly in step 1 with CMY-2, an abundant pAmpC beta-lactamase, but the formed acyl-intermediate did not noticeably hydrolyze in step 2, i.e., MEM acted as a potent inhibitor of the enzyme. This “antibiotic trapping” was confirmed by Goessens et al. (2013) with the detection of a covalent enzyme-MEM conjugate. On the other hand, the “trapping” by IPM is less momentous as its acyl intermediate hydrolyzes more rapidly.

Why does the CarbaLux test detect hyper-produced AmpCs (Table 3), whereas the prior pH methods cannot in spite of using the hydrolytically more reactive imipenem as substrate? A straightforward explanation is that the pH-based methods require a significant amount of hydrolyzed carbapenem substrate to shift the pH sufficiently for detection with a color indicator. This is not achievable with AmpCs. To support this hypothesis we determined the amount of acid required in the CNPt-direct for a change of color from red to orange in a titration experiment (Pasteran et al., 2015). When bacterial suspensions were investigated, the consumed acetic acid exceeded 30 times (based on molarities) the fluorescent carbapenem used in the CarbaLux test, thus explaining its higher selectivity.

However, this cannot explain the data of Table 4, collected from the three isolates Nos. 2, 16, and 79, which reflect considerably higher hydrolysis rates of IPM versus those of the fluorescent carbapenem. Neither does this agree with the high reactivity of the same isolates, found by the CarbaLux test, providing positive results already at room temperature. Therefore, an additional explanation was required.

Why is the CarbaLux test rapid in spite of the observed low hydrolysis rates of the fluorescent carbapenem? As observed in the inhibition assay, the retarded hydrolysis of nitrocefin revealed that the fluorescent carbapenem acted as a potent inhibitor of AmpC from isolate No. 79, reflecting its high affinity to the enzyme. With larger concentration of extracted enzymes and low substrate amounts, as investigated by the CarbaLux test, full inactivation of the substrate occurs already in step 1. The close structural relationship between MEM and the fluorescent carbapenem, together with their matching (IC_50_) inhibition properties and hydrolysis data (Table 4), strongly support the trapping mechanism. They additionally explain the rapid inactivation of the fluorescent substrate and the detection of particular beta-lactamases in the CarbaLux test. Similar considerations might be valid for some OXA carbapenemases, also having very high affinity to carbapenems and low catalytic efficiency (Walther-Rasmussen and Høiby, 2006; Santillana et al., 2007).

An important question arises about the origin of the beta-lactamases in the six CarbaLux positive isolates of Table 3. Are they over-expressed intrinsic chromosomal (cAmpC) enzymes or specific mutants thereof with enhanced activity towards carbapenems or acquired plasmid-mediated (pAmpC) enzymes? The phenotypic differentiation is particularly difficult in *K. aerogenes and E. cloacae* complex. The more so as high carbapenemase activity of AmpC strains was only scarcely reported. In general, plasmidic *bla*AmpCs are expressed constitutively (Polsfuss et al., 2011) and the enzymes are not inducible, although there are exceptions. The enzyme affinity of carbapenems is much higher (Queenan et al., 2010) and also the level of enzyme expression is also higher in comparison with the chromosomal *bla*AmpCs (Jacoby, 2009).

Based on the high carbapenemase activity, the lack of AmpC inducibility, and the agreement between the MEM inhibition activity of sonicated isolate No. 79 (IC_50_ 0.05 μM) and that of CMY-2 beta-lactamase (0.02 μM), (Goessens et al., 2013) we tentatively classify the isolate 79 as plasmidic *bla*AmpC carrier. However, further molecular investigations are needed for a more detailed characterization.

The simple CarbaLux test provides early information as to whether or not carbapenem antibiotics are an option for therapy. The investigation of viable bacteria from primary cultures adds to reliability. Being broader and less laborious, the test may be applied prior to molecular assays. Only when the phenotypic test was positive, would complementary genotypic investigations be necessary to determine which individual enzymes contribute to the resistance.

With regard to the protocols of all prior phenotypic tests, the fluorescence assay is the easiest to handle because predisposed vials with single portioned units (SPU) are available and because it does not require a double investigation of each tested strain with and without carbapenem substrate (control tube) as the pH based methods do. Up to ten tests can be carried out in parallel and with most carbapenemases a positive result is available within one hour. Reading of test results and interpretation are also easier and less ambiguous. Detecting classical carbapenemases and carbapenem inactivating AmpCs together in a single test is to the best advantage. Predisposed SPU test tubes, containing fluorescent carbapenem and inhibitor cloxacillin, allow investigating, whether AmpC was causative or contributed to a prior positive test result.

The mode of action of the CarbaLux test is straightforward and comprehensible in that the decay of the fluorescent carbapenem can be directly observed by vision under UV light and because high concentrations of enzymes and low amounts of substrate are investigated. These conditions are also present in the periplasm of bacteria (Gazouli et al., 1996) during therapy. Several consecutive readings allow estimating the reactivity of the extracted enzyme in each run. With particular bacteria, the test can proceed faster via a mechanism different from that of current rapid assays. It can also detect carbapenemase-producing bacteria, which might appear unsuspicious by antibiograms.

Due to the basic carbapenem structure of the fluorescent substrate, *the phenotypic* CarbaLux test reacts in the same way as carbapenem antibiotics do. It detects all carbapenem-deactivating Gram-negative bacteria, independent of their MICs, and/or the type and number of the relevant enzymes, the location of the genes (chromosomal or plasmidic), their mechanism of deactivation (trapping or hydrolysis). As cell extracts are investigated, permeability of the bacterial cell walls or the porin status does not influence the outcome of the test either. Without the necessity of targeting particular genes, the phenotypic CarbaLux test could serve as a first step for an assessment, aiming for all known carbapenemases and those to be detected in future.

A limitation can arise from initially unremarkable strains, which produce the resistance enzymes only during therapy. Repeated testing is recommended. The test should not exclude, but contribute to the therapist’s experience and knowledge, how to treat particular infections.

The European Committee on Antimicrobial Susceptibility Testing (EUCAST) recommends the same testing and infection control procedures for bacteria with pAmpC beta-lactamases as for classical carbapenemase carriers (EUCAST, 2017a). In the past, the incidence of AmpC carrying pathogens was hidden, not widely known, and a rapid and simple method of assessment was not available. Therefore, most clinical laboratories avoided investigation of this progressing resistance (Black et al., 2005) and still today, it goes undetected. Early and sustained implementation of the above guidelines may not only preserve patients and the community from the new threat but will also save enormous healthcare costs and be a blessing to modern medicine (Smith and Coast, 2013).

## Conclusion

The CarbaLux test was shown to be a reliable rapid test. It was safe, easy to handle and read in a routine diagnostic microbiological laboratory. Potentially, the new method could become a useful tool to investigate resistance mechanisms but also for measures of prescription practice and infection control. Eventually, it could also curtail the dissemination of multi-resistant Gram-negative pathogens by easier and broader detection.

## Data Availability Statement

The original contributions presented in the study are included in the article/supplementary material, further inquiries can be directed to the corresponding author.

## Ethics Statement

This study uses strains obtained from the collection of the Department of Medical Microbiology, München Klinik. The competent ethics committee of Ludwig Maximilian University confirmed that this study (Project No. 20-682 KB) did not require a review or approval by an ethics committee (https://www.med.uni-muenchen.de/ethik/beratungspflicht/index.html).

## Author Contributions

HP provided technical support and wrote the article. H-US directed and supervised the investigations. HF provided scientific expertise and institutional support. All authors contributed to the results and to conclusions of the study.

## Product Information

Materials are commercially available from carbalux.com.

## Conflict of Interest

HP was director of the company CarbaLux GmbH (carbalux.com). The remaining authors declare that the research was conducted in the absence of any commercial or financial relationships that could be construed as a potential conflict of interest.

## Patient and Public Involvement Statement

Patients and public were not involved in this study.
